# 

**Published:** 1983-07

**Authors:** 


					Contents
Editorial
97.
Ernest William Hey Groves and his
Contributions to Orthopaedic Surgery
Anthony H. C. Ratliff
98.
The First B.M.A. Meeting in Bristol
Patricia Craig
104.
The Venous Pump of the Human Foot?
Preliminary report
A. M. N. Gardner
109.
Collateral Pulmonary Respiration of
Van Allen
A. M. N. Gardner
113.
Carcinoma ot Prostate
A. M. N. Gardner and N. Setakis
117.
Aspects of General Practice Studied by
University of Bristol Students
Robin Philipp and Anthony Hughes
120.
Liver Hydatid Cyst Rupture
N. I. Ramus and T. Lyons
123.
Chronic Pancreatitis in Bristol
Sulieman S. Fedail, P. R. Salmon,
R. F. Harvey and A. E. Read
127.
Medicine and Birds
Philip Radford
130.
Obituary
?Sir Howard Middlemiss
133.
continued overleaf
100 Years Ago
136.
From Our Correspondents
139.
From Our Foreign Correspondent
144.
Centenary Dinner for the Bristol
Medico-Chirurgical Journal
148.
South West Orthopaedic Club
149.
Cossham Medical Society 1982-83
152.
153. List of Members

				

